# Outcomes of one-stage reconstruction for chronic multiligament injuries of knee

**DOI:** 10.1186/s43019-020-00083-y

**Published:** 2021-01-07

**Authors:** Tarun Goyal, Souvik Paul, Sushovan Banerjee, Lakshmana Das

**Affiliations:** 1 Department of Orthopaedics, All India Institute of Medical Sciences, Bathinda, Punjab 151001 India; 2 Department of Orthopaedics, All India Institute of Medical Sciences, Rishikesh, India

**Keywords:** Multiligament, Anterior cruciate ligament, Knee dislocation, Medial collateral ligament, Posterior cruciate ligament

## Abstract

**Purpose:**

This article aims to evaluate patterns of chronic multiligament injuries and outcomes of treatment with single-stage reconstruction using autografts.

**Methods:**

All patients with clinicoradiologically diagnosed multiligament knee injury (MKI) were included in this prospective observational study. As the time since injury was more than 6 weeks in all of the patients, they were categorized as having chronic MKI. Patients were assessed clinically for laxity, and the diagnosis was confirmed radiologically. Ipsilateral hamstring tendons were used for medial collateral ligament (MCL) or posterolateral corner reconstruction in a patient with Schenck knee dislocation (KD) type III. In these cases, the posterior cruciate ligament (PCL) and anterior cruciate ligament (ACL) were reconstructed by using the peroneus longus and contralateral hamstring tendons respectively. Ipsilateral hamstring tendons were used for ACL reconstruction and an ipsilateral peroneus longus tendon graft was used for reconstruction of the PCL in a KD type II injury. In two cases of KD type IV injury, the lateral laxity was only grade II and was managed conservatively; the rest of the ligaments were addressed like a KD type III injury. Outcome evaluation was done using a visual analogue scale (VAS) for pain, International Knee Documentation Committee (IKDC) score, Lysholm score, and Tegner activity level, preoperatively and postoperatively at 2 years’ follow-up.

**Results:**

A total of 27 patients of mean age 33.48 ± 9.9 years with MKI were included in the study. The patients were classified as eight KD type II, 17 KD type III, and two KD type IV. The majority of the patients had associated meniscal (59.2%) or chondral (40.7%) injuries. At the 2 years’ follow-up visit, there were significant improvements in VAS score (*p* = 0.0001) IKDC score (*p* = 0.0001), Lysholm score (*p* = 0.0001), and range of motion (*p* = 0.001). None of the patients had residual laxity on clinical examination of the knee joint at the 2 years’ follow-up. All but two of the patients went back to their previous activity level. These two patients had progressive knee arthritis and needed knee arthroplasty.

**Conclusion:**

Single-stage surgical reconstruction for chronic MKI has favourable functional outcomes.

**Level of evidence:**

Level IV, case series.

## Introduction

Multiligament injuries are serious injuries of the knee joint [1–5]. They are rare, contributing to approximately only 0.02–0.2% of all orthopaedic injuries [6]. A multiligament knee injury (MKI) can be defined as an injury to two or more major ligaments of the knee [2, 7]. Injuries more than 6 weeks old have been described as chronic injuries in the literature [8–11]. Chronic ligament injuries, particularly for the collateral ligaments, behave and are treated differently compared to acute injuries.

Management of these chronic injuries is controversial. Consensus is lacking on the use of staged or single-stage procedures, repair or reconstruction of ligaments, type of reconstruction for each ligament, the timing of surgery, graft options, the sequence of reconstruction, and postoperative rehabilitation [9]. Different treatment options have been proposed in different studies [2, 7, 10, 12–14]. These studies focus on the acute treatment of knee dislocation and multiligament injuries. However, most patients encountered in our setting had multiligament injuries more than 6 weeks old. When presented late, tissue repair is complicated by tissue retraction and fibrosis [11]. Allografts or allografts combined with autografts were used in all of the preceding studies. Allografts may have disadvantages like limited availability and risk of disease transmission. They may increase the costs of the surgery as well [15–18]. With many alternative autograft options available, reconstruction of multiple ligaments with autografts is possible.

This article aims to study outcomes of treatment of chronic MKI with single-stage reconstruction using autografts.

## Materials and methods

From July 2016 to June 2018, all patients with chronic MKI were included in this prospective observational study. Chronic MKI was defined as injuries 6 weeks old or more [9, 19, 20]. The diagnoses were made based on history, clinical examination, stress radiographs, and magnetic resonance imaging (MRI). Patients presenting with injury to two or more major ligaments: anterior cruciate ligament (ACL), posterior cruciate ligament (PCL), medial collateral ligament (MCL), and lateral collateral ligament (LCL), were included and classified according to the Schenck knee dislocation (KD) classification system [21]. Every patient was evaluated clinically for a possible vascular injury by palpation of distal pulses and ankle-brachial index (ABI). If there was a suspicion of a vascular compromise on this evaluation (ABI less than 0.90), a computed tomography (CT) angiography was performed. Limb malalignment was assessed by CT scanogram.

### Graft selection

Autograft tendons were used in all the reconstructions. Ipsilateral semitendinosus tendons were used for superficial medial collateral ligament (sMCL) and both semitendinosus and gracilis for posterolateral corner (PLC) reconstruction in a patient with classification KD type III. In these cases, the PCL and ACL were reconstructed with ipsilateral peroneus longus and contralateral hamstring tendon grafts respectively. Ipsilateral hamstring tendons were used for ACL reconstruction and an ipsilateral peroneus longus tendon graft was used for reconstruction of the PCL in a KD type II injury. In two cases of KD type IV injury, the lateral laxity was only grade II and was managed conservatively, and the rest of the ligament injuries were addressed similarly as for a KD type III injury.

### Surgical techniques

Patients were positioned supine on a standard orthopaedic table. Under spinal and epidural anaesthesia, the limb was draped after applying a well-padded high-thigh tourniquet and a side post. The opposite limb was also draped free for harvesting the tendon graft in patients with cases of KD types III and IV. All patients underwent intraoperative examination under anaesthesia, and an image intensifier was used to reach the final decision about the reconstruction of medial or lateral collateral ligaments.

**Fig. 1:**
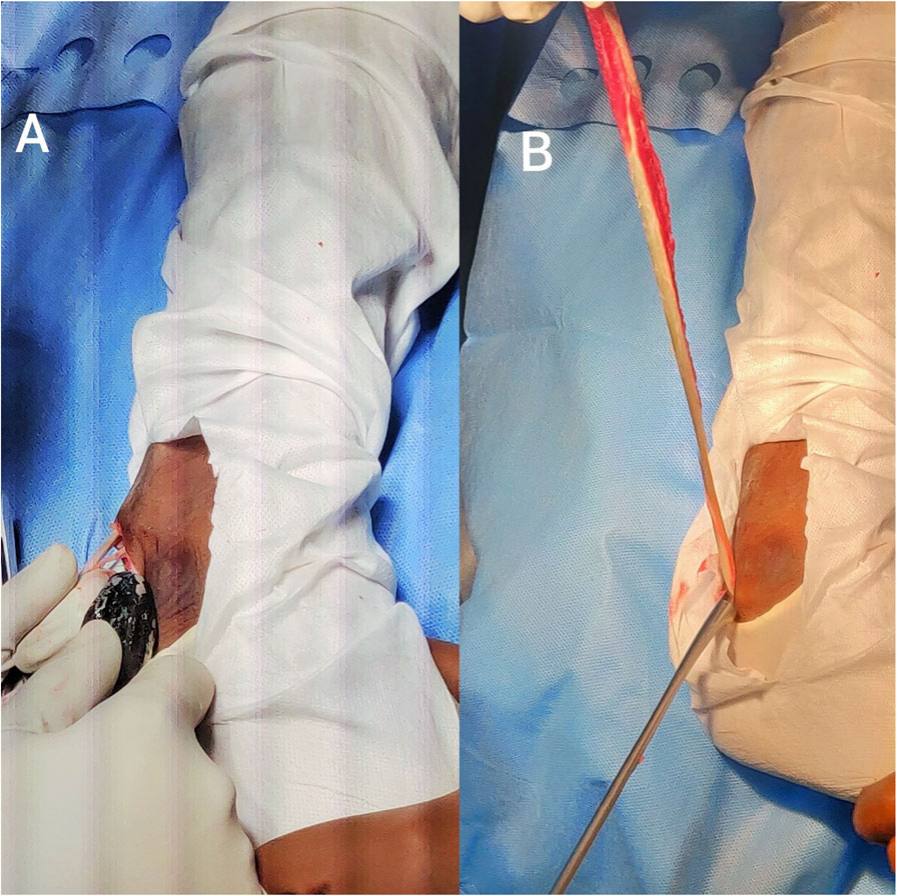
Depiction of **a** peroneus longus tendon being harvested using a tendon stripper and **b** harvested tendon attached distally

For harvesting the peroneus longus tendon, a 3-cm skin incision was made 1 cm behind the lateral malleolus. Any branches of the cutaneous nerve in this area were carefully protected. The peroneal tendons were identified after incising the superficial fascia and the superior peroneal retinaculum. The PLT was differentiated from the peroneus brevis tendon by its thicker size, its superficial location, and the absence of any muscle fibres attached to it. The PLT was marked and divided behind the lateral malleolus. The distal part of the tendon was stitched to the peroneus brevis tendon in end-to-side fashion. A whipstitch was made at the proximal free end of the PLT with an Ethibond No. 2 suture, and a closed tendon stripper was used to harvest the tendon (Fig. 1). The dimensions of the tendon graft were noted, and the tendon was prepared on a graft preparation board.

**Fig. 2:**
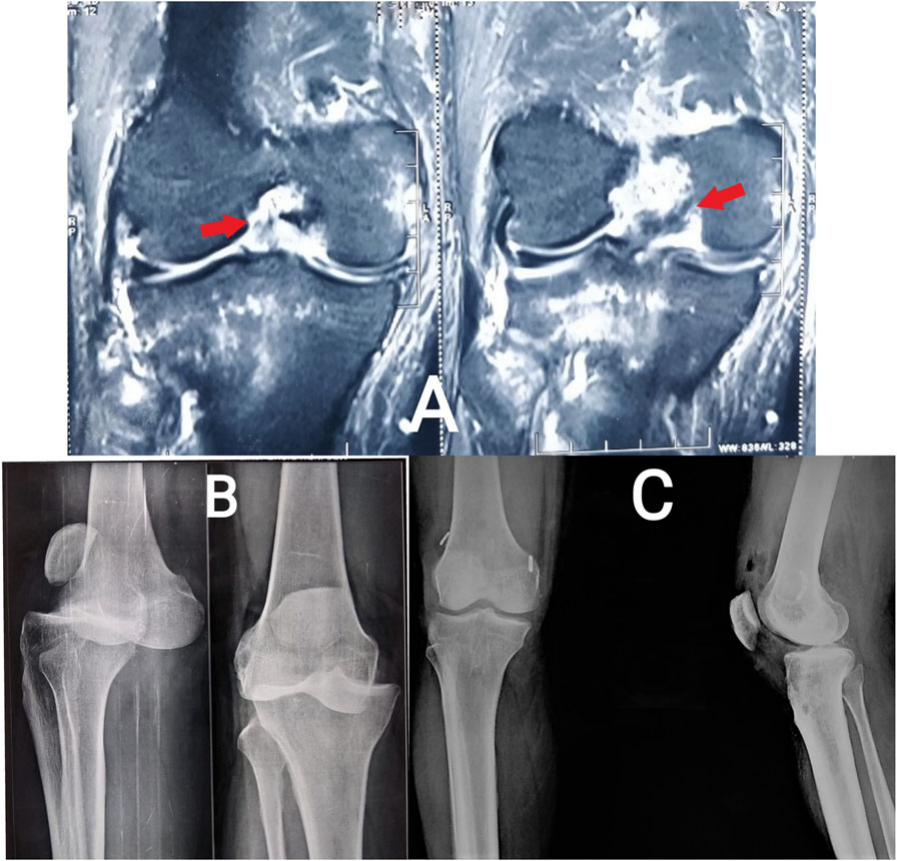
**a** Fat-suppressed coronal MRI images of a patient with ACL and PCL tears (*red arrows* show discontinuity of fibres from the femoral attachments). **b** For patient with a knee dislocation, radiographs before reduction of the knee joint. **c** For patient with a KD type II injury, postoperative radiograph after ACL and PCL reconstruction

**Fig. 3:**
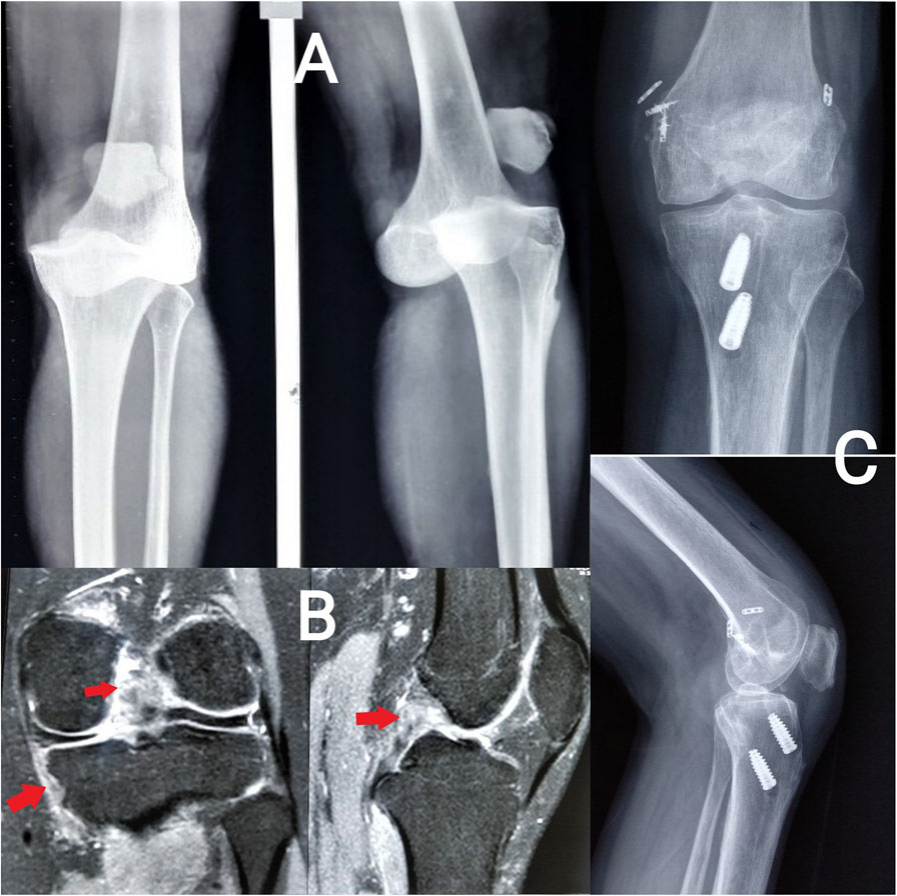
**a** Radiographs before reduction of the knee joint. **b** Fat-suppressed coronal and sagittal MRI images of a patient with ACL, PCL, and MCL tears. **c** For patient with a KD type III M injury, postoperative radiograph after ACL, PCL reconstruction and MCL repair

**Fig. 4:**
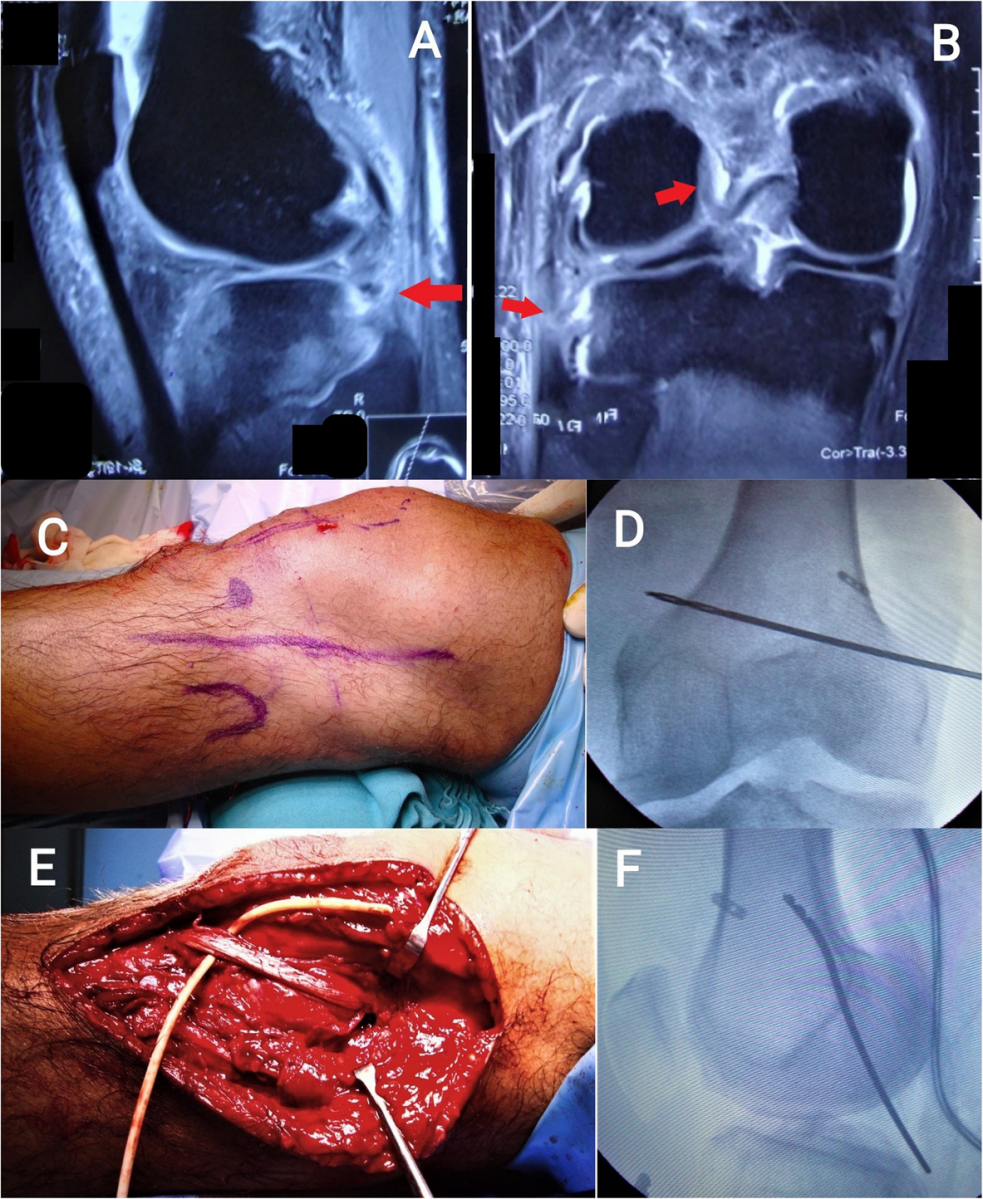
Fat-suppressed sagittal (**a**) and coronal (**b**) MRI images of a patient showing ACL and PLC injury tears (*red arrows* show discontinuity of ACL fibres from the femoral attachments and discontinuity of LCL and popliteus tendon). **c** Skin incision marked for reconstruction of PLC. **d**, **f** Intraoperative fluoroscopic images showing position of Beath pin in anteroposterior and lateral views. **e** Intraoperative clinical image showing semitendinosus tendon graft secured in the femur and the fibular head

**Fig. 5:**
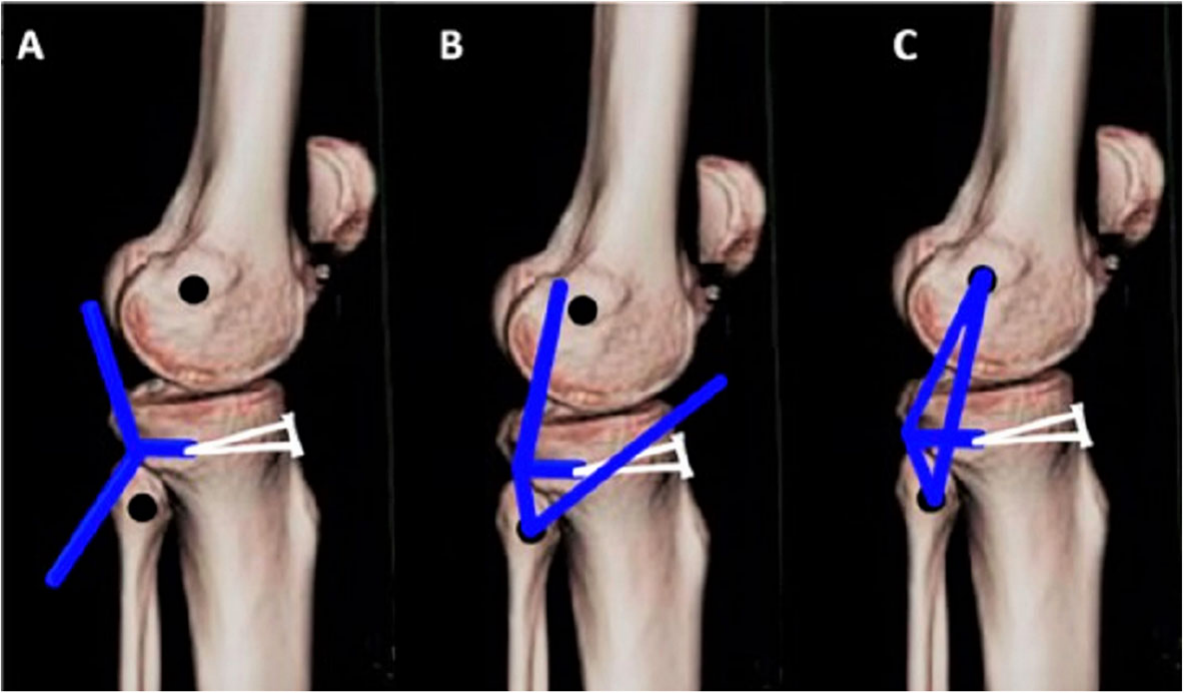
Technique of PLC reconstruction. **a** Semitendinosus tendon autograft (marked *blue*) loop fixed in tibial tunnel with suspensory device; **b** semitendinosus tendon autograft passed through fibular tunnel; **c** both strands of graft inserted into single femoral socket drilled at LCL insertion site

**Fig. 6:**
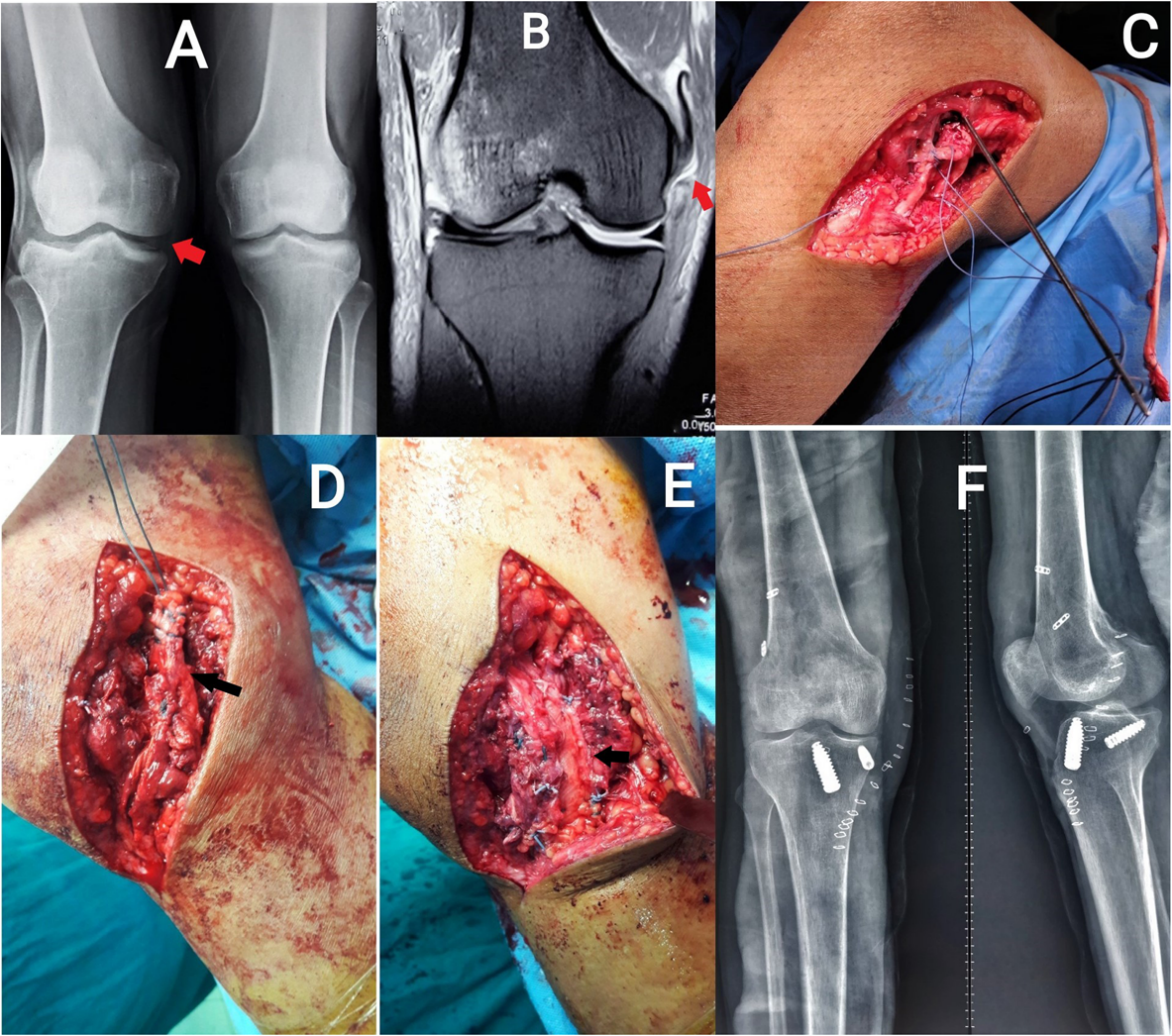
**a** Preoperative stress radiograph of bilateral knee showing opening of medial joint space of right knee more than left side, suggesting right medial collateral injury. **b** Preoperative MRI picture showing MCL injury. There is discontinuity of MCL with the distal part of the ligament retracted cephalad. **c** Intraoperative image showing Beath pin placement for drilling of femoral tunnel for MCL reconstruction. **d**, **e** Intraoperative pictures showing graft placement. The semitendinosus graft was left attached to the tibia distally and secured in the femoral tunnel. The free end of the graft was used for reconstruction of the POL. **f** Postoperative X-ray of the same patient showing fixation of the ACL, MCL, and POL with interference screws and adjustable-length suspensory button

Arthroscopic single-bundle ACL reconstruction was performed. Fixation on the femoral side was achieved using an adjustable suspensory device, and a bioabsorbable or metallic screw was used for tibial fixation. Arthroscopic single-bundle PCL reconstruction was performed; fixation on the femoral side was achieved with an adjustable suspensory device, and bioabsorbable screws were used for fixation on the tibial side (Figs. 2 and 3). For lateral-side injuries, anatomical posterolateral corner (PLC) reconstruction was performed with a semitendinosus tendon autograft [22]. The PLC reconstruction was performed using a single femoral socket for the LCL and popliteus, a tibial tunnel drilled from a point just distal and medial to Gerdy’s tubercle in an anterior-to-posterior direction, and a tunnel drilled anterolateral to the posteromedial direction starting at the insertion of the LCL to reach the fibular insertion of the popliteofibular ligament, similar to the technique described by LaPrade et al. [23]. The semitendinosus graft was first fixed in the tibial tunnel with adjustable loop fixation on the anterior cortex of the tibia. Then, as per the technique described by Franciozi et al. [22], the long arm of the semitendinosus graft was passed through the fibular tunnel. Both arms of the semitendinosus graft were fixed in the femoral socket with bioabsorbable or metallic screws (Figs. 4 and 5). On the medial side, reconstructions of the sMCL and posterior oblique ligament (POL) were performed using the ipsilateral semitendinosus tendon. Its attachment at the tibial insertion was left intact. On the femoral side, a loop of the grafts reconstructing both the sMCL and the POL were fixed together in the same tunnel with a suspensory device. The other end of the semitendinosus graft was used for reconstruction of the POL by fixing it at its anatomical insertion on the tibia with a bioabsorbable or metallic screw (Fig. 6).

The grafts were pretensioned on the graft board. All bone tunnels were drilled prior to passing the grafts inside the tunnels. The sequence of graft fixation was dependent on the type of injury. The PCL was fixed first while the posterior sag was corrected and the tension manually maintained at 70–90° of flexion. Thereafter, ACL fixation was performed at 20–30° of flexion with manual tensioning. The PLC and MCL were fixed at that point.

### Rehabilitation protocol

Isometric quadriceps exercises and ankle pumping exercises were started from the first postoperative day. All patients with collateral ligament reconstruction or meniscal repairs were kept non-weight bearing for 6 weeks, followed by partial weight bearing. Full weight-bearing mobilization started at 8 weeks with a long knee brace. A hinged range of motion knee brace was used for collateral ligament injuries for 6 weeks. Closed-chain knee range of motion was started up to 90° with a hinged knee brace after pain relief. Open-chain quadriceps and hamstring-strengthening exercises were started at 8 weeks. Patients were allowed to perform running and agility training after 9–12 months of physiotherapy.

All the patients were operated on by a single surgeon, and all were evaluated clinically for any laxity during the postoperative period. A follow-up functional outcome evaluation was performed using the visual analogue scale (VAS) for pain (0–10 cm scale), the International Knee Documentation Committee (IKDC) score, Lysholm score, and Tegner activity level, preoperatively and at 2 years’ follow-up by a single blinded observer. Institutional review board approval was obtained for the study. Informed consent was obtained from all the patients.

### Statistical analysis

The statistical analysis was done using SPSS 24.0. The data were studied for normality. Continuous variables were expressed as mean ± standard deviation. A paired *t* test was used to calculate the improvement between preoperative and postoperative functional outcome scores.

## Results

A total of 27 patients who presented with MKI were included in the study. There were 24 males and three females, with a mean age of 33.48 ± 9.9 years. Demographic details of the included patients are summarized in Table 1. Table 2 shows the distribution of clinical and functional outcome parameters. Table 3 presents the distribution of graft/tunnel diameters in reconstruction. Four patients (14.8%) were obese, and 12 patients (44.4%) were overweight. There were eight KD type II, 17 KD type III (11 III medial [M] and six III lateral [L]), and two KD type IV patients in this study. There were no patients with significant limb malalignment needing osteotomy. The majority of patients had associated meniscal or chondral injuries (Table 1). Meniscus tear was repaired in five patients (medial meniscus in four patients, lateral meniscus in one patient), and partial meniscectomy was performed in 11 patients (medial meniscus in seven patients, lateral meniscus in one patient, and both menisci in three patients). Outerbridge grade II osteochondral defects were managed by microfractures in six patients, and Outerbridge grade I osteochondral defects were left alone. Autologous osteochondral transfer was performed in two patients for an Outerbridge grade III lesion in the femoral condyle. Graft and tunnel diameters have been summarized in Table 3.

**Table 1:** Demographic parameters and activity levels of all patients (*n* = 27)

Variable	Results
Age in years (mean ± SD)	33.48 ± 9.9 (range 18–51)
Male, female	24, 3
Side	18 right, 9 left
BMI (mean ± SD)	26.7 ± 6.5 (range 20.8–32.5)
Mechanism of injury	22 road traffic accidents, 5 fall from height
Diagnosis	8 patients with ACL and PCL injury (KD II) 11 patients with ACL, PCL, and MCL injury (KD III M) 6 patients with ACL, PCL, PLC, and LCL injury (KD III L) 2 patients with ACL, PCL, MCL, and LCL injury (KD IV)
Duration since injury (mean ± SD)	14.6 ± 5.9 weeks (range 7–22 weeks)
Associated injuries	16 patients with meniscal injury (11 MM, 2 LM, 3 both menisci) 11 patients chondral damage (5 patients with OB grade I in femoral condyles, 3 patients OB grade I in tibial condyles, 3 patients with combined type I lesion in both condyles, 6 patients with OB grade II in femoral/tibial condyles, 3 patients with OB grade III changes in femoral condyle) 1 patient with popliteal artery injury
Preinjury activity level	3 patients Level 2 11 patients Level 3 7 patients Level 4 6 patients Level 5
Postinjury activity level	3 patients Level 2 11 patients Level 3 6 patients Level 4 5 patients Level 5

*BMI* body mass index, *ACL* anterior cruciate ligament, *PCL* posterior cruciate ligament, *KD* Schenck knee dislocation classification, *MCL* medial collateral ligament, *LCL* lateral collateral ligament, *PLC* posterolateral corner, *MM* medial meniscus, *LM* lateral meniscus, *OB* Outerbridge, *SD* standard deviation

**Table 2:** Distribution of clinical and functional outcome parameters

Variable	Mean ± standard deviation	Range	Significance
Knee range of flexion, unaffected side	127.1 ± 5.7	120–135	0.001
Knee range of flexion, affected side	111.5 ± 9.1	100–130	
Preoperative VAS score	7.4 ± 1.3	6–9	0.0001
Postoperative VAS score at 2 years’ follow-up	4.2 ± 1.5	2–6	
Preoperative Lysholm score	22.3 ± 9.7	6–38	0.0001
Postoperative Lysholm score at 2 years’ follow-up	50.41 ± 11.76	28–68	
Preoperative IKDC score	24.11 ± 5.08	14.3–32.9	0.0001
Postoperative IKDC score at 2 years’ follow-up	62.78 ± 5.05	53.7–71.3	

*VAS* visual analogue scale, *IKDC* International Knee Documentation Committee

**Table 3:** Distribution of graft and tunnel diameters (all measurements are in millimeters)

Graft/tunnel	ACL reconstruction	PCL reconstruction	MCL reconstruction	PLC reconstruction
Semitendinosus (ST) and gracilis (G)	Quadrupled ST + G: 8.68 ± 0.42		Double ST (sMCL): 6.9 ± 0.5	Double ST: 6.9 ± 0.5
Peroneus longus (double diameter)		8.3 ± 0.51		
Femoral tunnel	8 or 9	8 or 9	6, 7, or 8	LCL: 7 or 8 Popliteus: 7 or 8
Tibial tunnel	8, 9, or 10	8, 9, or 10	sMCL: 6 or 7 POL: 5 or 6	7 or 8
Fibular tunnel				6 or 7

*ACL* anterior cruciate ligament, *PCL* posterior cruciate ligament, *MCL* medial collateral ligament, *PLC* posterolateral corner, *sMCL* superficial medial collateral ligament, *LCL* lateral collateral ligament, *POL* posterior oblique ligament

None of the patients had residual grade II or III laxity in the knee on clinical examination at 2 years’ follow-up. Functional outcomes and range of motion had also improved significantly at 2 years’ follow-up (Table 2). None of the patients was a professional sportsperson. All but two of them had returned to their previous activity level by 2 years. Two of them had progressive knee arthritis and needed knee arthroplasty.

### Complications

Postoperative knee stiffness was observed in seven patients. Four patients improved with regular supervised physiotherapy. The rest of the patients improved after manipulation under anaesthesia. Two patients had superficial wound infections at the tibial fixation site which improved after oral antibiotics and superficial debridement. A total of six patients (four KD IV, two KD III) required CT angiography because of uncertain ABI results, and only one of them had associated popliteal artery injury; this was managed by popliteal artery bypass using a polytetrafluoroethylene graft by vascular surgeons followed by ligament reconstruction at a second stage. The patient had a persistent knee stiffness at 2 years’ follow-up, but vascularity of the lower limb was intact. Two patients (aged 46 and 48 years) with KD III injury underwent total knee arthroplasty due to progressive knee arthritis. Posterior stabilized knee prostheses were used in both patients.

## Discussion

The principal findings of this study are that there are satisfactory functional outcomes following single-stage surgical reconstruction with autografts in chronic MKI. Knowledge of management of chronic MKI is limited, and the majority of the literature discusses the management of acute injuries [2, 7, 9–11, 13, 24–26]. Most of the literature has considered less than 3 weeks as the cut-off duration for defining an acute injury, and more than 6 weeks as the time for chronic injuries [2, 9–11]. In this study, all of the patients presented after 6 weeks from injury with a mean duration of symptoms of 11.6 ± 4.9 weeks (range 7–22 weeks). The probable causes behind this may be associated with bony or neurovascular injuries or injuries being missed and treated with splints or being diagnosed as single ligament injury or delayed presentation from hilly terrains near our tertiary care centre. Outcomes of chronic MKI have been researched previously by only a few studies [20, 26–30]. Repair of the collateral ligaments, particularly the MCL, is complicated by scarring and soft tissue retraction in chronic cases [11]. Treatment decisions are complicated as the injury patterns are frequently diverse. It is difficult to assess and compare the outcomes because of the differences in treatment patterns. Nevertheless, this series describes the outcomes of single-stage multiligament reconstruction with autografts.

Surgery is the treatment of choice for MKI. Although studies before the year 1990 had favoured non-operative management [31, 32], recent developments in surgical techniques have brought a paradigm shift towards operative management of multiligament injuries [33, 34]. The timing of surgery in MKI has been a matter of debate. The recent literature favours early repair and reconstruction followed by aggressive physiotherapy [1, 7, 32, 35]. However, early surgery carries the risk of arthrofibrosis [11, 26, 36, 37].

Autograft options for ligament reconstruction are limited. Autografts have the advantage of early incorporation and easy availability, but there is a disadvantage of donor site morbidity [38, 39]. Allografts avoid these risks but are also associated with increased chances of infection and delayed incorporation, and they are neither cheap nor easily available [38, 40, 41]. Ipsilateral and contralateral hamstrings and ipsilateral peroneus longus tendons were used in this study. Contralateral hamstrings have been used by multiple authors in multiligament injuries with good results [41]. The peroneus longus is a useful and strong graft that can be used to reconstruct the PCL or ACL with minimum donor site morbidity [42–44]. The patellar bone tendon-bone graft or quadriceps tendon graft can also be harvested in these cases. These grafts are associated with increased chances of anterior knee pain or knee stiffness [41]. The authors did not encounter any graft fracture during the harvesting process. In cases of bicruciate reconstruction, if the graft size is smaller than expected, other autografts like peroneus longus, quadriceps tendon, or bone-patellar tendon-bone graft can be harvested from the ipsilateral or contralateral knee.

The sequence of tensioning of graft and tunnel management is another controversy in the management of MKI. The femoral tunnel convergence can be a problem when performing concomitant multiligament reconstructions. In a cadaveric study, Gelber at al [45]. suggested that optimal MCL and POL femoral tunnels should be both proximal and anterior at 30° coronal and 30° axial angulation to avoid collision with PCL tunnels. Tunnel convergence with the ACL tunnel has been found to occur at a rate up to 75% in an LCL tunnel of 30 mm depth and 69.4% in a 25-mm-deep tunnel [46]. The LCL can be drilled parallel to the distal condylar line and at an axial angle up to 40° anteriorly to avoid complications [46, 47]. The sequence of graft tensioning or ligament reconstruction is another debatable issue. In this study, the PCL was tensioned first, followed by the ACL, and the PLC or MCL was tensioned last. A biomechanical study by Moatshe et al. [48] revealed that tensioning the ACL first keeps the posterior sag uncorrected, and tensioning the PLC first produces excessive internal rotation of the tibia. This sequence has been followed before by different authors with satisfactory results [24, 26].

There is no consensus on a single-stage or two-stage ligament reconstruction. Systematic reviews by Hohmann et al. [49] and Levy et al. [9] have favoured single-stage early reconstruction. Mook et al. [50], Jiang et al. [51], and Ng et al. [2] have shown in their systematic reviews that better functional outcomes can be achieved with staged reconstruction. But most of these systematic reviews have included studies treating acute knee dislocation. In this study, single-stage reconstruction for all chronic multiligament injuries has achieved improved functional outcomes (Table 2).

Only a few case series have discussed the management and outcomes of chronic multigament injuries. Fanelli et al. [27] performed single-stage arthroscopic multiligament reconstruction of 10 acute and 10 chronic cases and followed them up for a mean of 24 months. There were one ACL/PCL tear, 10 ACL/PCL/PLC tears, seven ACL/PCL/MCL tears, and two ACL/PCL/MCL/PLC tears in this study. All of the patients had significant improvement (*p* = 0.0001) in their Lysholm, Tegner, and Hospital for Special Surgery knee scores postoperatively with no significant difference in functional outcomes between acute and chronic tears. Another study by Fanelli and Edson [28] included 19 patients with acute and 16 with chronic MKI (one ACL/PCL tear, 19 ACL/PCL/PLC tears, nine ACL/PCL/MCL tears, and six ACL/PCL/PLC/MCL tears). Significantly better functional outcome (*p* = 0.001) and lower translation measurements (*p* = 0.001) were noted after a minimum of 24 months follow-up. Karataglis et al. [29] studied six patients with acute and 29 patients with chronic MKI (mean duration since injury 2.7 years) for a mean of 40.3 months. Among these 35 patients, 28 were treated with arthroscopic single-stage reconstruction, and the rest were treated in two stages. A significant improvement in knee function with a mean knee flexion of 118.4° was noted. Noyes and Barber-Westin [30] performed single-stage femoral-fibular and cruciate reconstruction in 21 patients with combined injuries of posterolateral structures and cruciate ligament (ACL in 16, PCL in three, and ACL/PCL in two) at a mean duration of 2.7 years after the injury. Five patients among 21 had an early failure of femoral-fibular reconstruction, 2 to 29 months postoperatively. They could follow up 14 patients postoperatively to note normal to near-normal lateral joint opening and external rotation of the tibia in 76% of patients and significant improvement in Cincinnati Knee Rating System score (*p* < 0.0001) at 24 to 73 months’ follow-up. LaPrade et al. [20] compared 153 acute and 41 chronic multiligament injuries after single-stage arthroscopic reconstruction to reveal no differences in postoperative functional outcome scores. Reconstruction was favoured in all of these studies as repair had a high chance of failure [52]. Most of the previous studies have concentrated on the reconstruction of posterolateral corner (PLC) injuries associated with cruciate injuries. Moreover, allograft and autograft combinations were used in all these studies. The present study presents a variety of combinations of chronic multiligament injuries with a different combination of autografts. Autografts, if available, are biologically better than allografts. Allografts are commonly used in multiligament injuries as the dispensable sources of autograft have been considered limited. With more alternative options such as the peroneus longus tendon being used more frequently, autografts can be used for reconstruction of all these injuries.

The study was prospective in design; all surgeries were performed by a single surgeon, and findings were noted by a single blinded observer. The types of injuries and types of reconstruction were diverse, which made the assessment of outcomes difficult. The number of patients for each type of injury was relatively small. A comparative study on a larger scale and with a longer follow-up is required in the future.

## Conclusion

Multiligament injuries are challenging wounds that can be compounded by associated bony and neurovascular injuries. Single-stage surgical reconstruction for chronic cases has favourable outcomes in these injuries.

## Data Availability

The datasets used and/or analysed during the current study are available from the corresponding author on reasonable request.
